# Curcumin as a Modulator of Osteogenic Potential in Lipopolysaccharide-Treated Human Periodontal Ligament Cells: An In Vitro Study

**DOI:** 10.7759/cureus.76732

**Published:** 2025-01-01

**Authors:** Merita Stanley, Ravishankar PL, Prem Blaisie Rajula, Sunanda Rao, Priyankar Chakraborty, Amrutha Sairam

**Affiliations:** 1 Periodontology, SRM Kattankulathur Dental College and Hospital, SRM Institute of Science and Technology, Chennai, IND; 2 Periodontology, Agartala Government Dental College, Indira Gandhi Memorial Hospital, Tripura, IND

**Keywords:** anaerobic bacteria, bone morphogenetic protein 2, curcumin, inflammation, markers, osteopontin, periodontitis, regeneration, stem cells, tissue engineering

## Abstract

Background

Periodontal diseases cause alveolar bone destruction driven by anaerobic bacteria. Their virulence factors disrupt the osteogenic potential of human periodontal ligament stem cells (hPDLCs). Curcumin, a polyphenol with anti-inflammatory and osteogenic properties, holds promise for periodontal regeneration

Aim

This study evaluated the effects of curcumin on the osteogenic potential of hPDLCs under LPS-induced inflammatory conditions by assessing the expression of osteogenic markers, bone morphogenetic protein-2 (BMP-2), and osteopontin (OPN).

Materials and methods

hPDLCs were isolated from premolars and cultured in the presence of LPS (10 µg/mL) to simulate inflammation. Cells were treated with curcumin at 2.5 µM and 5 µM, with and without LPS exposure. Gene expression of BMP-2 and OPN was quantified using qRT-PCR after 21 days of culture.

Results

LPS significantly suppressed BMP-2 and OPN expression (*p* < 0.05). Curcumin treatment restored BMP-2 and OPN expression in a dose-dependent manner, with 5 µM curcumin demonstrating the most substantial effects, nearly restoring OPN levels to control values.

Conclusion

Curcumin mitigates LPS-induced inflammation and enhances hPDLCs' osteogenic differentiation, demonstrating its potential as a therapeutic adjunct for periodontal tissue regeneration.

## Introduction

Periodontal diseases impact up to 90% of the global population and are a major cause of tooth loss in adults, mainly resulting from the destruction of alveolar bone and supporting tissues [1]. The progression of the disease is driven by the buildup of subgingival plaque and the activity of gram-negative anaerobic bacteria, especially *Porphyromonas gingivalis*, a key pathogen in periodontal disease that disrupts the oral microbiome. A critical virulence factor of *P. gingivalis*, lipopolysaccharide (LPS), disrupts the periodontal microenvironment, damages periodontal tissue, reduces the viability of periodontal ligament stem cells, and impairs their differentiation. LPS also serves as a potent inflammatory mediator, inducing the release of pro-inflammatory cytokines and exacerbating tissue destruction [2].

A specialized subset of mesenchymal stem cells (MSCs), human periodontal ligament stem cells (hPDLSCs), has demonstrated immense potential in regenerative medicine. Their self-renewal ability, multipotency, and immunomodulatory properties make them particularly valuable for periodontal tissue engineering applications. hPDLSCs are especially promising for regenerating critical components of the periodontium [3,4]. During the repair process, they produce extracellular matrix proteins such as fibronectin, osteopontin, and fibromodulin while also increasing alkaline phosphatase activity to facilitate the formation of bone-like tissue [5].

Regenerating the periodontium is a complex process involving the formation of new cementum on exposed root surfaces, deposition of new alveolar bone, integration of functionally aligned connective tissue fibers into both the newly formed bone and cementum, and the restoration of healthy gingival structures [6]. Despite the availability of advanced treatments, including guided tissue regeneration, current periodontal therapies often fall short of fully restoring lost tissues [7]. This highlights the urgent need for innovative therapeutic strategies aimed at achieving comprehensive periodontal tissue regeneration [8].

Herbal remedies have long been integral to pharmaceutical development, and among these, curcumin, a polyphenol from *Curcuma longa*, has emerged as a compound with significant therapeutic potential. Curcumin improves bone mineral density and enhances bone microarchitecture, suggesting it may regulate bone remodeling by modulating osteocyte differentiation, either promoting or inhibiting it [9]. In addition, curcumin is well-known for its anti-inflammatory, antioxidant, and antimicrobial properties, highlighting its potential to address both soft and hard tissue complications in periodontal disease [10]. While various phytochemicals have been explored, no study has examined curcumin’s osteogenic potential in an inflammatory environment. This study investigates curcumin’s ability to enhance the osteogenic potential of human periodontal ligament cells (hPDLCs) under LPS-induced inflammation, highlighting its dual role in reducing inflammation and promoting periodontal tissue regeneration.

## Materials and methods

Study design

This in vitro study aimed to investigate the osteogenic effects of curcumin on hPDLCs under LPS-induced conditions. The primary objectives were to evaluate curcumin's potential to reduce LPS-induced inflammation and promote osteogenic differentiation by measuring the expression levels of bone morphogenetic protein-2 (BMP-2) and osteopontin (OPN).

The Institutional Ethical Committee Review Board of SRM Kattankulathur Dental College and Hospital accepted the study protocol on 21 September 2024 (SRMIEC-ST0924-1455).

Isolation and culture of hPDLCs

hPDLCs were isolated from healthy premolar teeth extracted for orthodontic treatment from individuals aged 12 to 18 years of both genders. Immediately after extraction, the teeth were disinfected with 1% penicillin-streptomycin in normal saline three times, followed by incubation for five minutes. PDL cells were scraped from the mid-third level of the root, and the tissue was incubated in a solution containing two digestive enzymes: 4 mg/mL dispase (GIBCO, Waltham, MA, USA) and 3 mg/mL collagenase type I (GIBCO, USA) for one hour at 37 °C. The cells were cultured in Dulbecco's Modified Eagle Medium (DMEM) supplemented with 1% penicillin-streptomycin, 1% L-glutamine, and 10% fetal bovine serum (FBS). The cultures were kept in a humidified incubator at 37°C with 5% CO₂, and cells from passages 3 to 5 were used for all experiments.

Formulation of curcumin for cell treatment

Curcumin (≥98% purity, Sigma-Aldrich) was first dissolved in dimethyl sulfoxide (DMSO) and then diluted with the culture medium to reach final concentrations of 2.5 µM and 5 µM.

Inflammation modeling and curcumin intervention

To simulate inflammatory conditions of periodontitis, hPDLCs were exposed to *Porphyromonas gingivalis*-derived LPS (10 μg/mL, sourced from Sigma-Aldrich, St. Louis, MO, USA) for 24 hours. Following this, cells were exposed to highly purified curcumin (≥98%, Sigma-Aldrich) at concentrations of 2.5 and 5.0 μM, with and without LPS exposure. All experimental procedures were conducted at 37°C and tested in triplicate.

Experimental groups

Cells were grown under four different conditions: For Group 1, hPDLC cells were cultured without LPS or curcumin (control group). For Group 2, hPDLC cells were cultured with LPS (10 µg/mL) to induce inflammation, which downregulates OPN and BMP-2 expression compared to the control (p < 0.05) (LPS group). For Group 3, hPDLC cells were pretreated with curcumin (2.5 µM and 5 µM) for two hours, followed by co-culture with LPS (LPS with curcumin group). For Group 4, hPDLC cells were treated with curcumin at 2.5 µM and 5 µM concentrations without LPS stimulation (curcumin group).

Gene expression analysis

To assess the molecular effects of curcumin on bone formation, the expression levels of key osteogenesis-related genes, BMP-2 and OPN, were analyzed using quantitative reverse-transcriptase polymerase chain reaction (qRT-PCR). RNA was extracted from all groups after 21 days of cell culture using TRIZOL reagent (Thermo Fisher Scientific, Waltham, MA, USA) following the manufacturer’s protocol. The quantity and purity of the RNA were assessed via UV spectrophotometry at 260 and 280 nm using a Nanodrop ND 100 (Thermo Fisher Scientific, Waltham, MA, USA).

Complementary DNA (cDNA) was synthesized from 500 ng of RNA using the Prime Script RT Reagent Kit (Perfect Real Time, TAKARA, Shiga, Japan) following the manufacturer's guidelines. Quantitative real-time PCR (qPCR) was performed with KAPA SYBR Fast Master Mix (2x) Universal (Kapa Biosystems, Sigma, Darmstadt, Germany) on the Bio-Rad Connect real-time PCR system (Bio-Rad, Hercules, CA, USA).

Two markers related to bone regeneration, OPN and BMP-2, were selected for analysis, with β-actin serving as the housekeeping gene for normalization to ensure consistent RNA levels across samples. Primers were designed using Primer-Blast software, as shown in Table 1, and the PCR amplification conditions were optimized for each primer pair. For both OPN and BMP-2, the PCR cycle began with an initial step at 95°C for three minutes, followed by 40 cycles at 95°C for three seconds. The final step lasted 30 seconds at 58°C for the former and 60°C for the latter. The run concluded with a dissociation curve, starting at 65°C for five seconds and gradually increasing to 95°C in 0.5°C increments. Data analysis was performed using Bio-Rad CFX Manager software version 3.0. The relative expression levels of BMP-2 and OPN were determined using the ΔΔCq method (where Cq represents the threshold cycle), with normalization to β-actin. Each experimental condition was performed in duplicate at the designated time points to ensure reliable results.

**Table 1:** Primer sequences for β-actin, OPN, and BMP-2

Gene	Forward 5’-3	Reverse 5’-3’
β-actin (housekeeping)	TCGTGTTGGATTCTGGGGAC	ACGAAGGAATAGCCACGCTC
OPN	CCTGGCTGAATTCTGAGGGAC	TATAGGATCTGGGTGCAGGCT
BMP-2	TGCTAGTAACTTTTGGCCATGATG	GCGTTTCCGCTGTTTGTGTT

The data analysis was conducted using IBM SPSS Statistics for Windows, Version 26.0 (released 2019, IBM Corp., Armonk, NY) with a comprehensive statistical approach. Descriptive statistics, including means and standard deviations, were calculated for each group. A one-way ANOVA was performed, followed by Tukey's HSD post-hoc test to detect statistically significant differences between groups, with statistical significance set at p < 0.05.

## Results

Table 2 outlines the effects of different concentrations of curcumin on osteopontin and bone morphogenetic protein-2 expression in hPDLCs exposed to LPS to induce inflammation.

**Table 2:** Effects of curcumin on osteopontin and BMP-2 expression in LPS-treated human periodontal ligament cells BMP-2: bone morphogenetic protein-2; LPS : lipopolysaccharide. One-way ANOVA; *p < 0.05.

Marker	Group	Mean	SD	p-value
Osteopontin expression	Control	1.000	0.000	0.007*
	LPS	0.421	0.107	
	LPS + Curcumin (2.5 µM)	0.667	0.211	
	LPS + Curcumin (5 µM)	1.078	0.273	
BMP-2 expression	Control	1.000	0.000	0.148
	LPS	0.265	0.189	
	LPS + Curcumin (2.5 µM)	0.800	0.365	
	LPS + Curcumin (5 µM)	1.019	0.678	

For osteopontin expression, the control group had a baseline mean value of 1.0. LPS treatment significantly reduced osteopontin expression to a mean of 0.42 ± 0.10 compared to the control (p = 0.007). When treated with curcumin at 2.5 µM and 5 µM concentrations, osteopontin levels increased to 0.667 ± 0.211 and 1.078 ± 0.273, respectively, indicating that curcumin may counteract the LPS-induced decrease in osteopontin expression. The findings suggest that curcumin, particularly at the 5 µM concentration, effectively counteracts the reduction in osteopontin expression caused by LPS exposure.

Table 2 shows the BMP-2 expression; the control group maintained a stable mean level of 1.0. When treated with LPS, BMP-2 expression was significantly reduced, with a mean of 0.26 ± 0.18, indicating that LPS exposure suppresses this critical bone morphogenetic protein. The introduction of curcumin at increasing concentrations led to a progressive increase in BMP-2 expression, with a mean of 0.8 ± 0.36 at 2.5 µM and 1.01 ± 0.67 at 5 µM. However, these increases in BMP-2 levels did not reach statistical significance (p = 0.148), suggesting that while curcumin may have a supportive role in enhancing BMP-2 expression following inflammatory insult, its effects require further investigation to confirm their efficacy and reproducibility.

**Figure 1:**
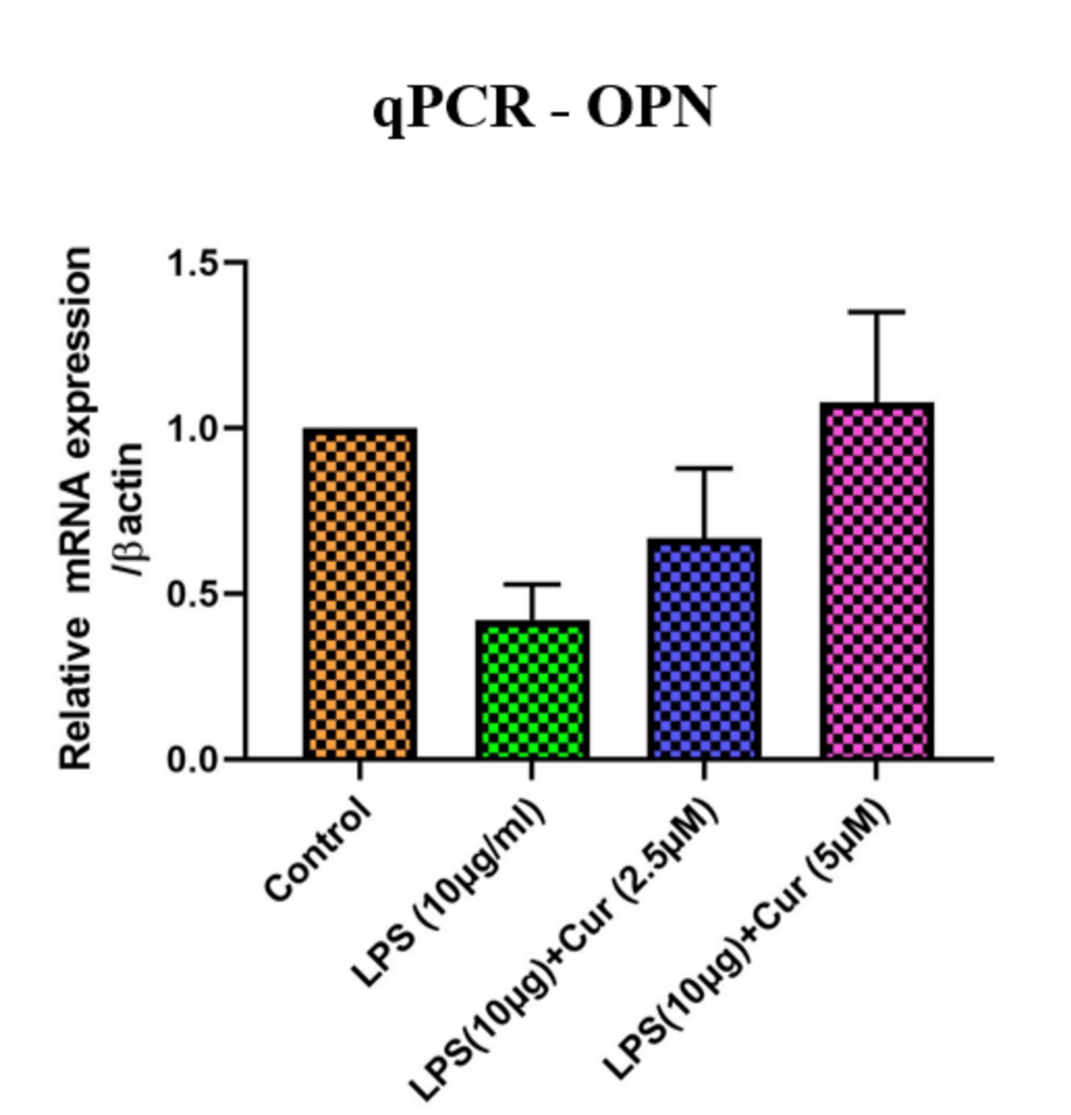
Effect of curcumin on OPN expression (qPCR nalysis) OPN: osteopontin

Figure 1 illustrates the relative mRNA expression for OPN, normalized to β-actin, across four treatment groups. The control group has the highest OPN expression. Treatment with LPS (10 μg/mL) alone significantly reduces OSP expression. Adding curcumin alongside LPS increases OPN expression in a dose-dependent manner, with 5 μM curcumin nearly restoring OPN levels compared to the control. This suggests that curcumin may counteract the LPS-induced decrease in OPN expression, with higher doses being more effective.

**Figure 2:**
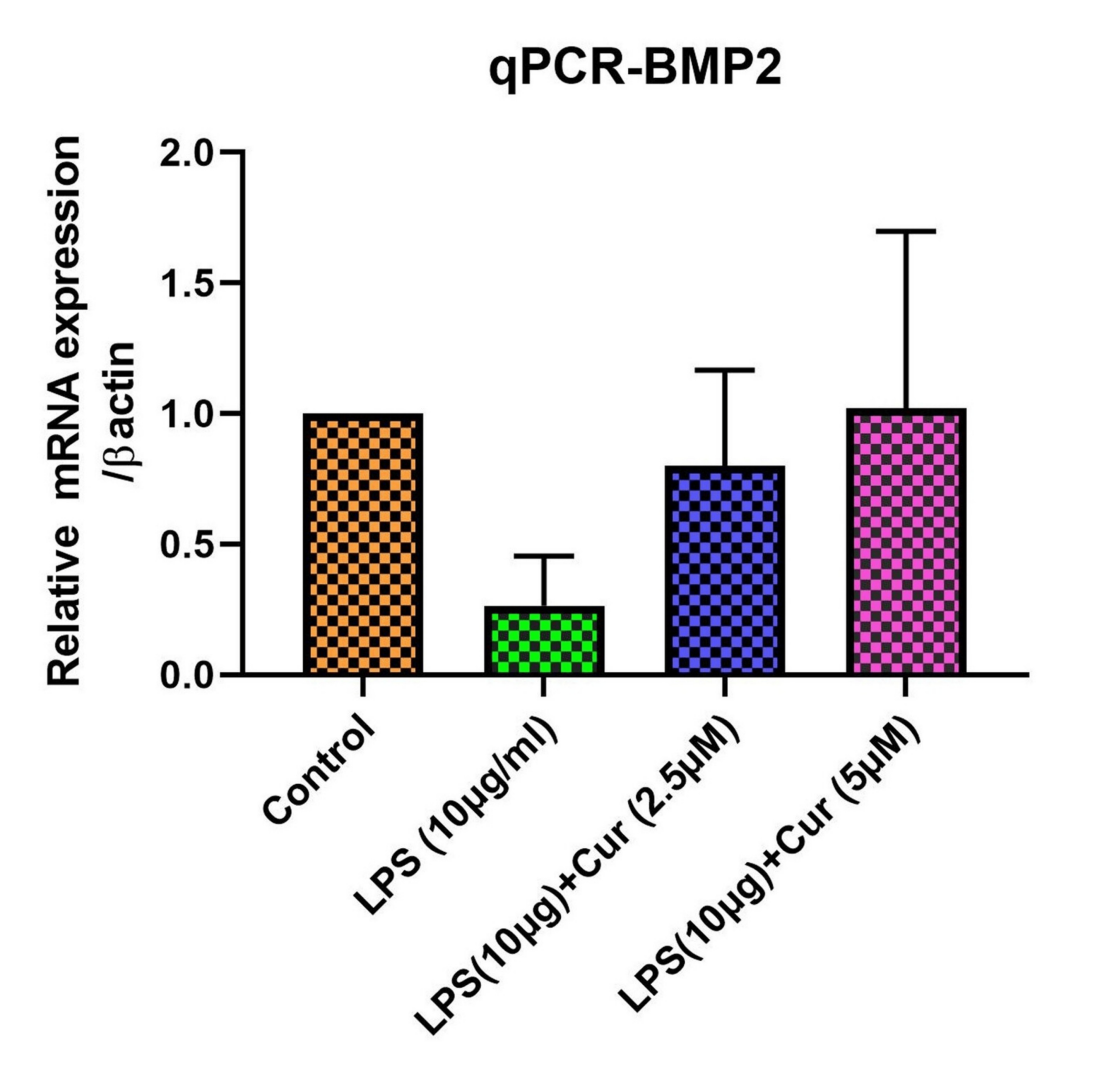
Curcumin's effect on BMP-2 expression (qPCR analysis) BMP-2: bone morphogenetic protein-2

Figure 2 shows the relative mRNA expression of BMP-2, normalized to β-actin, under different treatment conditions. The control group has a relatively high level of BMP-2 expression. Treatment with LPS (10 μg/mL) alone greatly reduces BMP-2 expression. When curcumin is added to LPS treatment, BMP-2 expression increases in a dose-dependent manner, with 5 μM curcumin resulting in a higher expression level than 2.5 μM. This suggests that curcumin may help counteract the LPS-induced decrease in BMP-2 expression, with higher concentrations showing a greater restorative effect.

Post-hoc comparisons of osteopontin expression revealed significant differences between the control and LPS groups, with a mean difference of 0.579 ± 0.018, indicating that LPS significantly decreased osteopontin levels. In addition, the LPS group differed significantly from the LPS + curcumin (5 µM) group, with a mean difference of 0.657 (p = 0.009), suggesting that 5 µM curcumin significantly restored osteopontin levels closer to the control. Comparisons between other groups, including between LPS + curcumin (2.5 µM) and LPS + curcumin (5 µM), showed no statistically significant differences (Table 3).

**Table 3:** Comparisons for osteopontin expression at various curcumin concentrations LPS: lipopolysaccharide. Tukey’s post-hoc test; *p < 0.05, **p < 0.01.

Comparison	Mean difference	p-value
Control vs. LPS	0.579	0.018*
Control vs. LPS + Curcumin (2.5 µM)	0.333	0.188
Control vs. LPS + Curcumin (5 µM)	0.078	0.950
LPS vs. LPS + Curcumin (2.5 µM)	0.246	0.397
LPS vs. LPS + Curcumin (5 µM)	0.657	0.009**
LPS + Curcumin (2.5 µM) vs. LPS + Curcumin (5 µM)	0.411	0.090

## Discussion

The primary goal of periodontal therapy is to regenerate the damaged tissues after successfully managing the inflammatory component of the disease [11]. Current clinical treatments for periodontitis, such as scaling, root planing, and surgical interventions, focus on controlling local inflammation and alleviating symptoms. While these approaches help prevent disease progression, they are limited in their ability to restore periodontal tissue attachment to teeth or regenerate the original periodontal structures [12].

To address these limitations, there is increasing interest in exploring novel, plant-derived natural compounds for regenerative purposes [13]. Among these, curcumin, a natural phytochemical, has drawn significant attention due to its extensive pharmacological properties and well-established safety profile. It promotes wound healing by promoting collagen deposition, granulation tissue formation, tissue remodeling, and wound contraction [14,15].

Bone homeostasis is regulated by the balance between bone formation and bone resorption, processes controlled by osteoblasts and osteoclasts, respectively [16]. This study evaluated the therapeutic potential of curcumin by examining its effects on two markers of periodontal health, BMP-2 and OPN, within an inflammatory environment induced by LPS to simulate periodontitis.

BMP-2, a member of the transforming growth factor-β superfamily, promotes the transition of MSCs into osteoblasts. In addition, BMPs can stimulate transcription and DNA synthesis associated with osteogenesis. They are also crucial in embryonic development and the early stages of fracture healing [17].

Osteopontin, a protein belonging to the small integrin-binding ligand N-linked glycoprotein family, is crucial for mineralized tissues and serves a vital function in the extracellular matrix of cementum and alveolar bone in the periodontal complex. It contributes to maintaining the structural integrity of the cementum-PDL-bone interfaces [18]. In addition, OPN is regarded as a late marker in the progression of these tissues [19].

The present study showed that LPS significantly suppressed BMP-2 and OPN expression compared to the control. Curcumin treatment restored BMP-2 levels dose-dependently, reaching 0.8 ± 0.36 at 2.5 µM and 1.01 ± 0.67 at 5 µM, and OPN levels increased to 0.667 ± 0.211 and 1.078 ± 0.273 at the same doses. These findings demonstrate curcumin's potential to counteract LPS-induced suppression of BMP-2 and OPN expression.

These results are consistent with a study by Tan et al., which highlighted curcumin's capability to restore the osteogenic capacity of human periodontal ligament stem cells under oxidative stress conditions induced by hydrogen peroxide [4]. Similarly, Pengjam et al. showed that a curcuminoid-rich extract in solid dispersion (CRE-SD) enhances osteoblast differentiation by upregulating BMP-2 and Runx2. This promotes osteogenic activity through BMP and Wnt/β-catenin signaling pathways, further supporting curcumin's potential as a therapeutic agent for bone-related conditions [20]. The study emphasizes curcumin's potential as a bioactive molecule for periodontal regeneration strategies.

The study's limitations include the need to explore a broader dose range to better understand dose-dependent effects and determine optimal therapeutic concentrations. Long-term investigations are required to evaluate curcumin's sustained impact on hPDLC differentiation and bone regeneration. Further studies should incorporate additional markers, such as Runx2, alkaline phosphatase, and COL1, to provide a more comprehensive understanding of curcumin's effects.

## Conclusions

The present study provides compelling evidence of curcumin's therapeutic potential in periodontal regeneration. By effectively mitigating the inflammatory environment induced by LPS and promoting the differentiation of hPDLCs toward the osteoblastic lineage, curcumin demonstrates its capacity to create a favorable microenvironment for both soft and hard tissue regeneration. However, to fully establish curcumin's clinical efficacy, further in vivo studies are essential. These studies will help determine optimal dosing, delivery strategies, and long-term outcomes, advancing curcumin as a potential treatment for periodontal therapy.
